# Working Memory From the Psychological and Neurosciences Perspectives: A Review

**DOI:** 10.3389/fpsyg.2018.00401

**Published:** 2018-03-27

**Authors:** Wen Jia Chai, Aini Ismafairus Abd Hamid, Jafri Malin Abdullah

**Affiliations:** ^1^Department of Neurosciences, School of Medical Sciences, Universiti Sains Malaysia, Kubang Kerian, Malaysia; ^2^Center for Neuroscience Services and Research, Universiti Sains Malaysia, Kubang Kerian, Malaysia

**Keywords:** working memory, neuroscience, psychology, cognition, brain, central executive, prefrontal cortex, review

## Abstract

Since the concept of working memory was introduced over 50 years ago, different schools of thought have offered different definitions for working memory based on the various cognitive domains that it encompasses. The general consensus regarding working memory supports the idea that working memory is extensively involved in goal-directed behaviors in which information must be retained and manipulated to ensure successful task execution. Before the emergence of other competing models, the concept of working memory was described by the multicomponent working memory model proposed by Baddeley and Hitch. In the present article, the authors provide an overview of several working memory-relevant studies in order to harmonize the findings of working memory from the neurosciences and psychological standpoints, especially after citing evidence from past studies of healthy, aging, diseased, and/or lesioned brains. In particular, the theoretical framework behind working memory, in which the related domains that are considered to play a part in different frameworks (such as memory’s capacity limit and temporary storage) are presented and discussed. From the neuroscience perspective, it has been established that working memory activates the fronto-parietal brain regions, including the prefrontal, cingulate, and parietal cortices. Recent studies have subsequently implicated the roles of subcortical regions (such as the midbrain and cerebellum) in working memory. Aging also appears to have modulatory effects on working memory; age interactions with emotion, caffeine and hormones appear to affect working memory performances at the neurobiological level. Moreover, working memory deficits are apparent in older individuals, who are susceptible to cognitive deterioration. Another younger population with working memory impairment consists of those with mental, developmental, and/or neurological disorders such as major depressive disorder and others. A less coherent and organized neural pattern has been consistently reported in these disadvantaged groups. Working memory of patients with traumatic brain injury was similarly affected and shown to have unusual neural activity (hyper- or hypoactivation) as a general observation. Decoding the underlying neural mechanisms of working memory helps support the current theoretical understandings concerning working memory, and at the same time provides insights into rehabilitation programs that target working memory impairments from neurophysiological or psychological aspects.

## Introduction

Working memory has fascinated scholars since its inception in the 1960’s (Baddeley, 2010; D’Esposito and Postle, 2015). Indeed, more than a century of scientific studies revolving around memory in the fields of psychology, biology, or neuroscience have not completely agreed upon a unified categorization of memory, especially in terms of its functions and mechanisms (Cowan, 2005, 2008; Baddeley, 2010). From the coining of the term “memory” in the 1880’s by Hermann Ebbinghaus, to the distinction made between primary and secondary memory by William James in 1890, and to the now widely accepted and used categorizations of memory that include: short-term, long-term, and working memories, studies that have tried to decode and understand this abstract concept called memory have been extensive (Cowan, 2005, 2008). Short and long-term memory suggest that the difference between the two lies in the period that the encoded information is retained. Other than that, long-term memory has been unanimously understood as a huge reserve of knowledge about past events, and its existence in a functioning human being is without dispute (Cowan, 2008). Further categorizations of long-term memory include several categories: (1) episodic; (2) semantic; (3) Pavlovian; and (4) procedural memory (Humphreys et al., 1989). For example, understanding and using language in reading and writing demonstrates long-term storage of semantics. Meanwhile, short-term memory was defined as temporarily accessible information that has a limited storage time (Cowan, 2008). Holding a string of meaningless numbers in the mind for brief delays reflects this short-term component of memory. Thus, the concept of working memory that shares similarities with short-term memory but attempts to address the oversimplification of short-term memory by introducing the role of information manipulation has emerged (Baddeley, 2012). This article seeks to present an up-to-date introductory overview of the realm of working memory by outlining several working memory studies from the psychological and neurosciences perspectives in an effort to refine and unite the scientific knowledge concerning working memory.

## The Multicomponent Working Memory Model

When one describes working memory, the multicomponent working memory model is undeniably one of the most prominent working memory models that is widely cited in literatures (Baars and Franklin, 2003; Cowan, 2005; Chein et al., 2011; Ashkenazi et al., 2013; D’Esposito and Postle, 2015; Kim et al., 2015). Baddeley and Hitch (1974) proposed a working memory model that revolutionized the rigid and dichotomous view of memory as being short or long-term, although the term “working memory” was first introduced by Miller et al. (1960). The working memory model posited that as opposed to the simplistic functions of short-term memory in providing short-term storage of information, working memory is a multicomponent system that manipulates information storage for greater and more complex cognitive utility (Baddeley and Hitch, 1974; Baddeley, 1996, 2000b). The three subcomponents involved are phonological loop (or the verbal working memory), visuospatial sketchpad (the visual-spatial working memory), and the central executive which involves the attentional control system (Baddeley and Hitch, 1974; Baddeley, 2000b). It was not until 2000 that another component termed “episodic buffer” was introduced into this working memory model (Baddeley, 2000a). Episodic buffer was regarded as a temporary storage system that modulates and integrates different sensory information (Baddeley, 2000a). In short, the central executive functions as the “control center” that oversees manipulation, recall, and processing of information (non-verbal or verbal) for meaningful functions such as decision-making, problem-solving or even manuscript writing. In Baddeley and Hitch (1974)’s well-cited paper, information received during the engagement of working memory can also be transferred to long-term storage. Instead of seeing working memory as merely an extension and a useful version of short-term memory, it appears to be more closely related to activated long-term memory, as suggested by Cowan (2005, 2008), who emphasized the role of attention in working memory; his conjectures were later supported by Baddeley (2010). Following this, the current development of the multicomponent working memory model could be retrieved from Baddeley’s article titled “Working Memory” published in *Current Biology*, in Figure 2 (Baddeley, 2010).

## An Embedded-Processes Model of Working Memory

**FIGURE 1:**
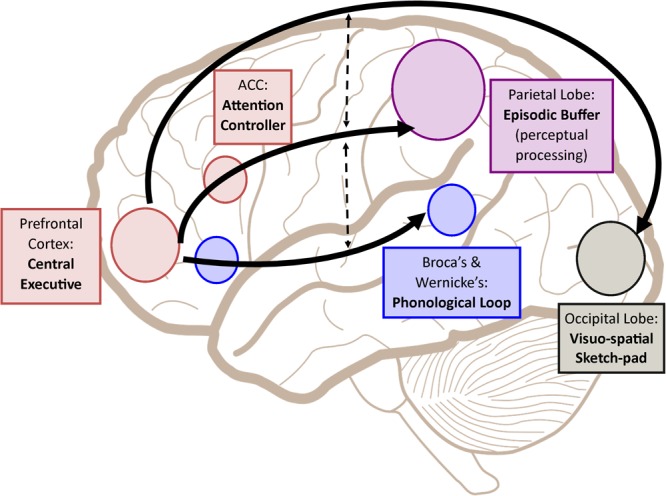
A simplified depiction (adapted from the multicomponent working memory model by Baddeley, 2010) as implicated in the brain, in which the central executive assumes the role to exert control and oversee the manipulation of incoming information for intended execution. ACC, Anterior cingulate cortex.

Notwithstanding the widespread use of the multicomponent working memory model, Cowan (1999, 2005) proposed the embedded-processes model that highlights the roles of long-term memory and attention in facilitating working memory functioning. Arguing that the Baddeley and Hitch (1974) model simplified perceptual processing of information presentation to the working memory store without considering the focus of attention to the stimuli presented, Cowan (2005, 2010) stressed the pivotal and central roles of working memory capacity for understanding the working memory concept. According to Cowan (2008), working memory can be conceptualized as a short-term storage component with a capacity limit that is heavily dependent on attention and other central executive processes that make use of stored information or that interact with long-term memory. The relationships between short-term, long-term, and working memory could be presented in a hierarchical manner whereby in the domain of long-term memory, there exists an intermediate subset of activated long-term memory (also the short-term storage component) and working memory belongs to the subset of activated long-term memory that is being attended to (Cowan, 1999, 2008). An illustration of Cowan’s theoretical framework on working memory can be traced back to Figure 1 in his paper titled “What are the differences between long-term, short-term, and working memory?” published in *Progress in Brain Research* (Cowan, 2008).

## Alternative Models

Cowan’s theoretical framework toward working memory is consistent with Engle (2002)’s view, in which it was posited that working memory capacity is comparable to directed or held attention information inhibition. Indeed, in their classic study on reading span and reading comprehension, Daneman and Carpenter (1980) demonstrated that working memory capacity, which was believed to be reflected by the reading span task, strongly correlated with various comprehension tests. Surely, recent and continual growth in the memory field has also demonstrated the development of other models such as the time-based resource-sharing model proposed by several researchers (Barrouillet et al., 2004, 2009; Barrouillet and Camos, 2007). This model similarly demonstrated that cognitive load and working memory capacity that were so often discussed by working memory researchers were mainly a product of attention that one receives to allocate to tasks at hand (Barrouillet et al., 2004, 2009; Barrouillet and Camos, 2007). In fact, the allocated cognitive resources for a task (such as provided attention) and the duration of such allocation dictated the likelihood of success in performing the tasks (Barrouillet et al., 2004, 2009; Barrouillet and Camos, 2007). This further highlighted the significance of working memory in comparison with short-term memory in that, although information retained during working memory is not as long-lasting as long-term memory, it is not the same and deviates from short-term memory for it involves higher-order processing and executive cognitive controls that are not observed in short-term memory. A more detailed presentation of other relevant working memory models that shared similar foundations with Cowan’s and emphasized the roles of long-term memory can be found in the review article by (D’Esposito and Postle, 2015).

In addition, in order to understand and compare similarities and disparities in different proposed models, about 20 years ago, Miyake and Shah (1999) suggested theoretical questions to authors of different models in their book on working memory models. The answers to these questions and presentations of models by these authors gave rise to a comprehensive definition of working memory proposed by Miyake and Shah (1999, p. 450), “working memory is those mechanisms or processes that are involved in the control, regulation, and active maintenance of task-relevant information in the service of complex cognition, including novel as well as familiar, skilled tasks. It consists of a set of processes and mechanisms and is not a fixed ‘place’ or ‘box’ in the cognitive architecture. It is not a completely unitary system in the sense that it involves multiple representational codes and/or different subsystems. Its capacity limits reflect multiple factors and may even be an emergent property of the multiple processes and mechanisms involved. Working memory is closely linked to LTM, and its contents consist primarily of currently activated LTM representations, but can also extend to LTM representations that are closely linked to activated retrieval cues and, hence, can be quickly activated.” That said, in spite of the variability and differences that have been observed following the rapid expansion of working memory understanding and its range of models since the inception of the multicomponent working memory model, it is worth highlighting that the roles of executive processes involved in working memory are indisputable, irrespective of whether different components exist. Such notion is well-supported as Miyake and Shah, at the time of documenting the volume back in the 1990’s, similarly noted that the mechanisms of executive control were being heavily investigated and emphasized (Miyake and Shah, 1999). In particular, several domains of working memory such as the focus of attention (Cowan, 1999, 2008), inhibitory controls (Engle and Kane, 2004), maintenance, manipulation, and updating of information (Baddeley, 2000a, 2010), capacity limits (Cowan, 2005), and episodic buffer (Baddeley, 2000a) were executive processes that relied on executive control efficacy (see also Miyake and Shah, 1999; Barrouillet et al., 2004; D’Esposito and Postle, 2015).

## The Neuroscience Perspective

Following such cognitive conceptualization of working memory developed more than four decades ago, numerous studies have intended to tackle this fascinating working memory using various means such as decoding its existence at the neuronal level and/or proposing different theoretical models in terms of neuronal activity or brain activation patterns. **Table 1** offers the summarized findings of these literatures. From the cognitive neuroscientific standpoint, for example, the verbal and visual-spatial working memories were examined separately, and the distinction between the two forms was documented through studies of patients with overt impairment in short-term storage for different verbal or visual tasks (Baddeley, 2000b). Based on these findings, associations or dissociations with the different systems of working memory (such as phonological loops and visuospatial sketchpad) were then made (Baddeley, 2000b). It has been established that verbal and acoustic information activates Broca’s and Wernicke’s areas while visuospatial information is represented in the right hemisphere (Baddeley, 2000b). Not surprisingly, many supporting research studies have pointed to the fronto-parietal network involving the dorsolateral prefrontal cortex (DLPFC), the anterior cingulate cortex (ACC), and the parietal cortex (PAR) as the working memory neural network (Osaka et al., 2003; Owen et al., 2005; Chein et al., 2011; Kim et al., 2015). More precisely, the DLPFC has been largely implicated in tasks demanding executive control such as those requiring integration of information for decision-making (Kim et al., 2015; Jimura et al., 2017), maintenance and manipulation/retrieval of stored information or relating to taxing loads (such as capacity limit) (Osaka et al., 2003; Moore et al., 2013; Vartanian et al., 2013; Rodriguez Merzagora et al., 2014), and information updating (Murty et al., 2011). Meanwhile, the ACC has been shown to act as an “attention controller” that evaluates the needs for adjustment and adaptation of received information based on task demands (Osaka et al., 2003), and the PAR has been regarded as the “workspace” for sensory or perceptual processing (Owen et al., 2005; Andersen and Cui, 2009). **Figure 1** attempted to translate the theoretical formulation of the multicomponent working memory model (Baddeley, 2010) to specific regions in the human brain. It is, however, to be acknowledged that the current neuroscientific understanding on working memory adopted that working memory, like other cognitive systems, involves the functional integration of the brain as a whole; and to clearly delineate its roles into multiple components with only a few regions serving as specific buffers was deemed impractical (D’Esposito and Postle, 2015). Nonetheless, depicting the multicomponent working memory model in the brain offers a glimpse into the functional segregation of working memory.

**Table 1:** Working memory (WM) studies in the healthy brain.

Authors	WM Components	WM Task	Neuroimaging Modality	Brain Regions Involved
Bolkan et al., 2017 (animal study: mice)	WM maintenance	Spatial WM task (T-maze)	–	MD, medial PFC
Chein et al., 2011	WM storage and processing/recall	Complex WM span task	fMRI	PFC, ACC, PPC, MTL
Jimura et al., 2017	Cognitive control; WM load	Intertemporal decision-making task; Sternberg WM task	fMRI	anterior PFC, DLPFC, IFJ, pre-SMA, AI, PPC, tempo-parietal junction
Kim et al., 2015	Information integration	Arithmetic task	fMRI	IFG, MFG, FPC, DLPFC
Moore et al., 2013	WM encoding, maintenance, and retrieval	Verbal WM task	fMRI	MFG, IFS, DLPFC, caudate, thalamus, parietal and cingulate regions
Murty et al., 2011	Selective updating	Digital-updating WM task	fMRI	DLPFC, caudate, SN/VTA, parietal, cerebellar, and cingulate regions
Osaka et al., 2003	WM capacity	Verbal WM task	fMRI	PFC, ACC, STG
Vartanian et al., 2013	WM capacity	N-back task; AUT	fMRI	DLPFC, VLPFC, anterior PFC, OFC, SMA

*AUT, Alternate Uses Task; fMRI, Functional magnetic resonance imaging; TMS, Transcranial magnetic stimulation; SMA, Supplementary motor area; IFG, Inferior frontal gyrus; FEF, Frontal eye field; AI, Anterior insula; IFJ, Inferior frontal junctions; DLPFC, Dorsolateral prefrontal cortex; MD, Mediodorsal thalamus; PFC, Prefrontal cortex; ACC, Anterior cingulate cortex; PPC, Posterior parietal cortex; MTL, Medial temporal lobe; MFG, Middle frontal gyrus; FPC, Frontopolar cortex; PCS, Precentral sulcus; IPS, Intraparietal sulcus; IFS, Inferior frontal sulcus; SN/VTA, Substantia nigra and ventral tegmental area; STG, Superior temporal gyrus; VLPFC, Ventrolateral prefrontal cortex; OFC, Orbitofrontal cortex.*

Further investigation has recently revealed that other than the generally informed cortical structures involved in verbal working memory, basal ganglia, which lies in the subcortical layer, plays a role too (Moore et al., 2013). Particularly, the caudate and thalamus were activated during task encoding, and the medial thalamus during the maintenance phase, while recorded activity in the fronto-parietal network, which includes the DLPFC and the parietal lobules, was observed only during retrieval (Moore et al., 2013). These findings support the notion that the basal ganglia functions to enhance focusing on a target while at the same time suppressing irrelevant distractors during verbal working memory tasks, which is especially crucial at the encoding phase (Moore et al., 2013). Besides, a study conducted on mice yielded a similar conclusion in which the mediodorsal thalamus aided the medial prefrontal cortex in the maintenance of working memory (Bolkan et al., 2017). In another study by Murty et al. (2011) in which information updating, which is one of the important aspects of working memory, was investigated, the midbrain including the substantia nigra/ventral tegmental area and caudate was activated together with DLPFC and other parietal regions. Taken together, these studies indicated that brain activation of working memory are not only limited to the cortical layer (Murty et al., 2011; Moore et al., 2013). In fact, studies on cerebellar lesions subsequently discovered that patients suffered from impairments in attention-related working memory or executive functions, suggesting that in spite of the motor functions widely attributed to the cerebellum, the cerebellum is also involved in higher-order cognitive functions including working memory (Gottwald et al., 2004; Ziemus et al., 2007).

Shifting the attention to the neuronal network involved in working memory, effective connectivity analysis during engagement of a working memory task reinforced the idea that the DLPFC, PAR and ACC belong to the working memory circuitry, and bidirectional endogenous connections between all these regions were observed in which the left and right PAR were the modeled input regions (Dima et al., 2014) (refer to Supplementary Figure 1 in Dima et al., 2014). Effective connectivity describes the attempt to model causal influence of neuronal connections in order to better understand the hidden neuronal states underlying detected neuronal responses (Friston et al., 2013). Another similar study of working memory using an effective connectivity analysis that involved more brain regions, including the bilateral middle frontal gyrus (MFG), ACC, inferior frontal cortex (IFC), and posterior parietal cortex (PPC) established the modulatory effect of working memory load in this fronto-parietal network with memory delay as the driving input to the bilateral PPC (Ma et al., 2012) (refer to Figure 1 in Ma et al., 2012).

Moving away from brain regions activated but toward the in-depth neurobiological side of working memory, it has long been understood that the limited capacity of working memory and its transient nature, which are considered two of the defining characteristics of working memory, indicate the role of persistent neuronal firing (see Review Article by D’Esposito and Postle, 2015; Zylberberg and Strowbridge, 2017; see also Silvanto, 2017), that is, continuous action potentials are generated in neurons along the neural network. However, this view was challenged when activity-silent synaptic mechanisms were found to also be involved (Mongillo et al., 2008; Rose et al., 2016; see also Silvanto, 2017). Instead of holding relevant information through heightened and persistent neuronal firing, residual calcium at the presynaptic terminals was suggested to have mediated the working memory process (Mongillo et al., 2008). This synaptic theory was further supported when TMS application produced a reactivation effect of past information that was not needed or attended at the conscious level, hence the TMS application facilitated working memory efficacy (Rose et al., 2016). As it happens, this provided evidence from the neurobiological viewpoint to support Cowan’s theorized idea of “activated long-term memory” being a feature of working memory as non-cued past items in working memory that were assumed to be no longer accessible were actually stored in a latent state and could be brought back into consciousness. However, the researchers cautioned the use of the term “activated long-term memory” and opted for “prioritized long-term memory” because these unattended items maintained in working memory seemed to employ a different mechanism than items that were dropped from working memory (Rose et al., 2016). Other than the synaptic theory, the spiking working memory model proposed by Fiebig and Lansner (2017) that borrowed the concept from fast Hebbian plasticity similarly disagreed with persistent neuronal activity and demonstrated that working memory processes were instead manifested in discrete oscillatory bursts.

## Age and Working Memory

Nevertheless, having established a clear working memory circuitry in the brain, differences in brain activations, neural patterns or working memory performances are still apparent in different study groups, especially in those with diseased or aging brains. For a start, it is well understood that working memory declines with age (Hedden and Gabrieli, 2004; Ziaei et al., 2017). Hence, older participants are expected to perform poorer on a working memory task when making comparison with relatively younger task takers. In fact, it was reported that decreases in cortical surface area in the frontal lobe of the right hemisphere was associated with poorer performers (Nissim et al., 2017). In their study, healthy (those without mild cognitive impairments [MCI] or neurodegenerative diseases such as dementia or Alzheimer’s) elderly people with an average age of 70 took the n-back working memory task while magnetic resonance imaging (MRI) scans were obtained from them (Nissim et al., 2017). The outcomes exhibited that a decrease in cortical surface areas in the superior frontal gyrus, pars opercularis of the inferior frontal gyrus, and medial orbital frontal gyrus that was lateralized to the right hemisphere, was significantly detected among low performers, implying an association between loss of brain structural integrity and working memory performance (Nissim et al., 2017). There was no observed significant decline in cortical thickness of the studied brains, which is assumed to implicate neurodegenerative tissue loss (Nissim et al., 2017).

Moreover, another extensive study that examined cognitive functions of participants across the lifespan using functional magnetic resonance imaging (fMRI) reported that the right lateralized fronto-parietal regions in addition to the ventromedial prefrontal cortex (VMPFC), posterior cingulate cortex, and left angular and middle frontal gyri (the default mode regions) in older adults showed reduced modulation of task difficulty, which was reflective of poorer task performance (Rieck et al., 2017). In particular, older-age adults (55–69 years) exhibited diminished brain activations (positive modulation) as compared to middle-age adults (35–54 years) with increasing task difficulty, whereas lesser deactivation (negative modulation) was observed between the transition from younger adults (20–34 years) to middle-age adults (Rieck et al., 2017). This provided insights on cognitive function differences during an individual’s lifespan at the neurobiological level, which hinted at the reduced ability or efficacy of the brain to modulate functional regions to increased difficulty as one grows old (Rieck et al., 2017). As a matter of fact, such an opinion was in line with the Compensation-Related Utilization of Neural Circuits Hypothesis (CRUNCH) proposed by Reuter-Lorenz and Cappell (2008). The CRUNCH likewise agreed upon reduced neural efficiency in older adults and contended that age-associated cognitive decline brought over-activation as a compensatory mechanism; yet, a shift would occur as task loads increase and under-activation would then be expected because older adults with relatively lesser cognitive resources would max out their ‘cognitive reserve’ sooner than younger adults (Reuter-Lorenz and Park, 2010; Schneider-Garces et al., 2010).

In addition to those findings, emotional distractors presented during a working memory task were shown to alter or affect task performance in older adults (Oren et al., 2017; Ziaei et al., 2017). Based on the study by Oren et al. (2017) who utilized the n-back task paired with emotional distractors with neutral or negative valence in the background, negative distractors with low load (such as 1-back) resulted in shorter response time (RT) in the older participants (*M*_age_ = 71.8), although their responses were not significantly more accurate when neutral distractors were shown. Also, lesser activations in the bilateral MFG, VMPFC, and left PAR were reported in the old-age group during negative low load condition. This finding subsequently demonstrated the results of emotional effects on working memory performance in older adults (Oren et al., 2017). Further functional connectivity analyses revealed that the amygdala, the region well-known to be involved in emotional processing, was deactivated and displayed similar strength in functional connectivity regardless of emotional or load conditions in the old-age group (Oren et al., 2017). This finding went in the opposite direction of that observed in the younger group in which the amygdala was strongly activated with less functional connections to the bilateral MFG and left PAR (Oren et al., 2017). This might explain the shorter reported RT, which was an indication of improved working memory performance, during the emotional working memory task in the older adults as their amygdala activation was suppressed as compared to the younger adults (Oren et al., 2017).

Interestingly, a contrasting neural connection outcome was reported in the study by Ziaei et al. (2017) in which differential functional networks relating to emotional working memory task were employed by the two studied groups: (1) younger (*M*_age_ = 22.6) and (2) older (*M*_age_ = 68.2) adults. In the study, emotional distractors with positive, neutral, and negative valence were presented during a visual working memory task and older adults were reported to adopt two distinct networks involving the VMPFC to encode and process positive and negative distractors while younger adults engaged only one neural pathway (Ziaei et al., 2017). The role of amygdala engagement in processing only negative items in the younger adults, but both negative and positive distractors in the older adults, could be reflective of the older adults’ better ability at regulating negative emotions which might subsequently provide a better platform for monitoring working memory performance and efficacy as compared to their younger counterparts (Ziaei et al., 2017). This study’s findings contradict those by Oren et al. (2017) in which the amygdala was found to play a bigger role in emotional working memory tasks among older participants as opposed to being suppressed as reported by Oren et al. (2017). Nonetheless, after overlooking the underlying neural mechanism relating to emotional distractors, it was still agreed that effective emotional processing sustained working memory performance among older/elderly people (Oren et al., 2017; Ziaei et al., 2017).

Aside from the interaction effect between emotion and aging on working memory, the impact of caffeine was also investigated among elders susceptible to age-related cognitive decline; and those reporting subtle cognitive deterioration 18-months after baseline measurement showed less marked effects of caffeine in the right hemisphere, unlike those with either intact cognitive ability or MCI (Haller et al., 2017). It was concluded that while caffeine’s effects were more pronounced in MCI participants, elders in the early stages of cognitive decline displayed diminished sensitivity to caffeine after being tested with the n-back task during fMRI acquisition (Haller et al., 2017). It is, however, to be noted that the working memory performance of those displaying minimal cognitive deterioration was maintained even though their brain imaging uncovered weaker brain activation in a more restricted area (Haller et al., 2017). Of great interest, such results might present a useful brain-based marker that can be used to identify possible age-related cognitive decline.

Similar findings that demonstrated more pronounced effects of caffeine on elderly participants were reported in an older study, whereas older participants in the age range of 50–65 years old exhibited better working memory performance that offset the cognitive decline observed in those with no caffeine consumption, in addition to displaying shorter reaction times and better motor speeds than observed in those without caffeine (Rees et al., 1999). Animal studies using mice showed replication of these results in mutated mice models of Alzheimer’s disease or older albino mice, both possibly due to the reported results of reduced amyloid production or brain-derived neurotrophic factor and tyrosine-kinase receptor. These mice performed significantly better after caffeine treatment in tasks that supposedly tapped into working memory or cognitive functions (Arendash et al., 2006). Such direct effects of caffeine on working memory in relation to age was further supported by neuroimaging studies (Haller et al., 2013; Klaassen et al., 2013). fMRI uncovered increased brain activation in regions or networks of working memory, including the fronto-parietal network or the prefrontal cortex in old-aged (Haller et al., 2013) or middle-aged adults (Klaassen et al., 2013), even though the behavioral measures of working memory did not differ. Taken together, these outcomes offered insight at the neurobiological level in which caffeine acts as a psychoactive agent that introduces changes and alters the aging brain’s biological environment that explicit behavioral testing might fail to capture due to performance maintenance (Haller et al., 2013, 2017; Klaassen et al., 2013).

With respect to physiological effects on cognitive functions (such as effects of caffeine on brain physiology), estradiol, the primary female sex hormone that regulates menstrual cycles, was found to also modulate working memory by engaging different brain activity patterns during different phases of the menstrual cycle (Joseph et al., 2012). The late follicular (LF) phase of the menstrual cycle, characterized by high estradiol levels, was shown to recruit more of the right hemisphere that was associated with improved working memory performance than did the early follicular (EF) phase, which has lower estradiol levels although overall, the direct association between estradiol levels and working memory was inconclusive (Joseph et al., 2012). The finding that estradiol levels modified brain recruitment patterns at the neurobiological level, which could indirectly affect working memory performance, presents implications that working memory impairment reported in post-menopausal women (older aged women) could indicate a link with estradiol loss (Joseph et al., 2012). In 2000, post-menopausal women undergoing hormone replacement therapy, specifically estrogen, were found to have better working memory performance in comparison with women who took estrogen and progestin or women who did not receive the therapy (Duff and Hampson, 2000). Yet, interestingly, a study by Janowsky et al. (2000) showed that testosterone supplementation counteracted age-related working memory decline in older males, but a similar effect was not detected in older females who were supplemented with estrogen. A relatively recent paper might have provided the explanation to such contradicting outcomes (Schöning et al., 2007). As demonstrated in the study using fMRI, the nature of the task (such as verbal or visual-spatial) might have played a role as a higher level of testosterone (in males) correlated with activations of the left inferior parietal cortex, which was deemed a key region in spatial processing that subsequently brought on better performance in a mental-rotation task. In contrast, significant correlation between estradiol and other cortical activations in females in the midluteal phase, who had higher estradiol levels, did not result in better performance of the task compared to women in the EF phase or men (Schöning et al., 2007). Nonetheless, it remains premature to conclude that age-related cognitive decline was a result of hormonal (estradiol or testosterone) fluctuations although hormones might have modulated the effect of aging on working memory.

Other than the presented interaction effects of age and emotions, caffeine, and hormones, other studies looked at working memory training in the older population in order to investigate working memory malleability in the aging brain. Findings of improved performance for the same working memory task after training were consistent across studies (Dahlin et al., 2008; Borella et al., 2017; Guye and von Bastian, 2017; Heinzel et al., 2017). Such positive results demonstrated effective training gains regardless of age difference that could even be maintained until 18 months later (Dahlin et al., 2008) even though the transfer effects of such training to other working memory tasks need to be further elucidated as strong evidence of transfer with medium to large effect size is lacking (Dahlin et al., 2008; Guye and von Bastian, 2017; Heinzel et al., 2017; see also Karbach and Verhaeghen, 2014). The studies showcasing the effectiveness of working memory training presented a useful cognitive intervention that could partially stall or delay cognitive decline. **Table 2** presents an overview of the age-related working memory studies.

**Table 2:** Working memory (WM) studies in relation to age.

Authors	Target groups	WM task	Neuroimaging modality	Outcome variables
Arendash et al., 2006 (animal study: Mice)	Heterozygous male mice carrying the mutant APP_K670N, M671L_ gene (APPsw)	Radial Arm Water Maze	–	Behavioral task performances
Borella et al., 2017	Old (*M*_age_ = 68.5)	CWMS task; LST; The Jigsaw Puzzle test – Puzzle	–	Neuropsychological test performances after training
Dahlin et al., 2008	Old (*M*_age_ = 68.3)	Computation span task; Forward and backward digit span (WAIS); N-back task	–	Neuropsychological test performances after training
Duff and Hampson, 2000	Post-menopausal women (*M*_age_ = 55.7)	Digit-ordering task; Spatial working-memory task	–	Neuropsychological test performances
Guye and von Bastian, 2017	Old (*M*_age_ = 70.2)	Complex span task; binding task; memory updating task	–	Neuropsychological test performances after training
Haller et al., 2017	Subtle cognitive deterioration; Mild cognitive impairments	N-back task	fMRI	Functional connectivity after caffeine intake
Haller et al., 2013	Old (*M*_age_ = 68.8)	N-back task	fMRI; MRI	Brain activations in ROIs; Functional connectivity; Baseline perfusion
Joseph et al., 2012	Pre-menopausal women	N-back task	fMRI	Brain activations in ROIs
Klaassen et al., 2013	Middle-aged (*M*_age_ = 49.2)	Letter Sternberg task	fMRI	Brain activations in ROIs
Nissim et al., 2017	Old (*M*_age_ = 70.3)	N-back task	MRI	Cortical surface area; cortical thickness
Oren et al., 2017	Old (*M*_age_ = 71.8)	Emotional n-back task	fMRI	Functional connectivity
Rees et al., 1999	Young- (*M*_age_ = 23.5) and middle-aged (*M*_age_ = 56.5)	Digit span task	–	Neuropsychological test performances
Rieck et al., 2017	Across the lifespan (age = 20–94)	Spatial distance judgment task	fMRI	Brain activations to task difficulty
Ziaei et al., 2017	Old (*M*_age_ = 68.2)	Visual WM task with emotional distractors	fMRI	Functional connectivity

*APPsw, Amyloid precursor protein, Swedish; CWMS, Categorization working memory span; LST, Listening span test; WAIS, Wechsler Adult Intelligence Scale; fMRI, Functional magnetic resonance imaging; MRI, Magnetic resonance imaging; ROIs, Regions of interest.*

## The Diseased Brain and Working Memory

Age is not the only factor influencing working memory. In recent studies, working memory deficits in populations with mental or neurological disorders were also being investigated (see **Table 3**). Having identified that the working memory circuitry involves the fronto-parietal region, especially the prefrontal and parietal cortices, in a healthy functioning brain, targeting these areas in order to understand how working memory is affected in a diseased brain might provide an explanation for the underlying deficits observed at the behavioral level. For example, it was found that individuals with generalized or social anxiety disorder exhibited reduced DLPFC activation that translated to poorer n-back task performance in terms of accuracy and RT when compared with the controls (Balderston et al., 2017). Also, VMPFC and ACC, representing the default mode network (DMN), were less inhibited in these individuals, indicating that cognitive resources might have been divided and resulted in working memory deficits due to the failure to disengage attention from persistent anxiety-related thoughts (Balderston et al., 2017). Similar speculation can be made about individuals with schizophrenia. Observed working memory deficits might be traced back to impairments in the neural networks that govern attentional-control and information manipulation and maintenance (Grot et al., 2017). The participants performed a working memory binding task, whereby they had to make sure that the word-ellipse pairs presented during the retrieval phase were identical to those in the encoding phase in terms of location and verbal information; results concluded that participants with schizophrenia had an overall poorer performance compared to healthy controls when they were asked to actively bind verbal and spatial information (Grot et al., 2017). This was reflected in the diminished activation in the schizophrenia group’s ventrolateral prefrontal cortex and the PPC that were said to play a role in manipulation and reorganization of information during encoding and maintenance of information after encoding (Grot et al., 2017).

**Table 3:** Working memory (WM) studies in the diseased brain.

Authors	Target groups	WM task	Neuroimaging modality	Outcome variables
Ashkenazi et al., 2013	Mathematical disabilities	WMTB-C	fMRI	Brain activations in ROIs
Balderston et al., 2017	Generalized/Social anxiety disorder	N-back WM task	fMRI	Brain activations in ROIs
Grot et al., 2017	Schizophrenia	WM binding task	fMRI	Brain activations in ROIs
Le et al., 2017	MDD	Delayed recognition task	fMRI	Functional connectivity
Maehler and Schuchardt, 2016	Dyslexia; dyscalculia; ADHD	Phonological, visuospatial, and central executive tasks	–	Neuropsychological test performances
Rotzer et al., 2009	Developmental dyscalculia	Corsi Block Tapping test	fMRI	Brain activations in ROIs
Stegmayer et al., 2015	Bipolar affective disorder	Verbal delayed matching to sample task	fMRI	Functional connectivity
Wang and Gathercole, 2013	Reading difficulties	AWMA	–	Neuropsychological test performances

*ADHD, Attention deficit/hyperactivity disorder; MDD, Major depressive disorder; WMTB-C, Working memory test battery for children; VSAT, Visual Serial Addition Task; AWMA, Automated Working Memory Assessment; fMRI, Functional magnetic resonance imaging; ROIs, Regions of interest.*

In addition, patients with major depressive disorder (MDD) displayed weaker performance in the working memory updating domain in which information manipulation was needed when completing a visual working memory task (Le et al., 2017). The working memory task employed in the study was a delayed recognition task that required participants to remember and recognize the faces or scenes as informed after stimuli presentation while undergoing fMRI scan (Le et al., 2017). Subsequent functional connectivity analyses revealed that the fusiform face area (FFA), parahippocampal place area (PPA), and left MFG showed aberrant activity in the MDD group as compared to the control group (Le et al., 2017). These brain regions are known to be the visual association area and the control center of working memory and have been implicated in visual working memory updating in healthy adults (Le et al., 2017). Therefore, altered visual cortical functions and load-related activation in the prefrontal cortex in the MDD group implied that the cognitive control for visual information processing and updating might be impaired at the input or control level, which could have ultimately played a part in the depressive symptoms (Le et al., 2017).

Similarly, during a verbal delayed match to sample task that asked participants to sub-articulatorly rehearse presented target letters for subsequent letter-matching, individuals with bipolar affective disorder displayed aberrant neural interactions between the right amygdala, which is part of the limbic system implicated in emotional processing as previously described, and ipsilateral cortical regions often concerned with verbal working memory, pointing out that the cortico-amygdalar connectivity was disrupted, which led to verbal working memory deficits (Stegmayer et al., 2015). As an attempt to gather insights into previously reported hyperactivation in the amygdala in bipolar affective disorder during an articulatory working memory task, functional connectivity analyses revealed that negative functional interactions seen in healthy controls were not replicated in patients with bipolar affective disorder (Stegmayer et al., 2015). Consistent with the previously described study about emotional processing effects on working memory in older adults, this reported outcome was suggestive of the brain’s failed attempts to suppress pathological amygdalar activation during a verbal working memory task (Stegmayer et al., 2015).

Another affected group with working memory deficits that has been the subject of research interest was children with developmental disorders such as attention deficit/hyperactivity disorder (ADHD), developmental dyscalculia, and reading difficulties (Rotzer et al., 2009; Ashkenazi et al., 2013; Wang and Gathercole, 2013; Maehler and Schuchardt, 2016). For instance, looking into the different working memory subsystems based on Baddeley’s multicomponent working memory model in children with dyslexia and/or ADHD and children with dyscalculia and/or ADHD through a series of tests, it was reported that distinctive working memory deficits by groups could be detected such that phonological loop (e.g., digit span) impairment was observed in the dyslexia group, visuospatial sketchpad (e.g., Corsi block tasks) deficits in the dyscalculia group, while central executive (e.g., complex counting span) deficits in children with ADHD (Maehler and Schuchardt, 2016). Meanwhile, examination of working memory impairment in a delayed match-to-sample visual task that put emphasis on the maintenance phase of working memory by examining the brainwaves of adults with ADHD using electroencephalography (EEG) also revealed a marginally significantly lower alpha band power in the posterior regions as compared to healthy individuals, and such an observation was not significantly improved after working memory training (Cogmed working memory training, CWMT Program) (Liu et al., 2016). The alpha power was considered important in the maintenance of working memory items; and lower working memory accuracy paired with lower alpha band power was indeed observed in the ADHD group (Liu et al., 2016).

Not dismissing the above compiled results, children encountering disabilities in mathematical operations likewise indicated deficits in the working memory domain that were traceable to unusual brain activities at the neurobiological level (Rotzer et al., 2009; Ashkenazi et al., 2013). It was speculated that visuospatial working memory plays a vital role when arithmetic problem-solving is involved in order to ensure intact mental representations of the numerical information (Rotzer et al., 2009). Indeed, Ashkenazi et al. (2013) revealed that Block Recall, a variant of the Corsi Block Tapping test and a subtest of the Working Memory Test Battery for Children (WMTB-C) that explored visuospatial sketchpad ability, was significantly predictive of math abilities. In relation to this, studies investigating brain activation patterns and performance of visuospatial working memory task in children with mathematical disabilities identified the intraparietal sulcus (IPS), in conjunction with other regions in the prefrontal and parietal cortices, to have less activation when visuospatial working memory was deemed involved (during an adapted form of Corsi Block Tapping test made suitable for fMRI [Rotzer et al., 2009]); in contrast the control group demonstrated correlations of the IPS in addition to the fronto-parietal cortical activation with the task (Rotzer et al., 2009; Ashkenazi et al., 2013). These brain activity variations that translated to differences in overt performances between healthily developing individuals and those with atypical development highlighted the need for intervention and attention for the disadvantaged groups.

## Traumatic Brain Injury and Working Memory

Physical injuries impacting the frontal or parietal lobes would reasonably be damaging to one’s working memory. This is supported in studies employing neuropsychological testing to assess cognitive impairments in patients with traumatic brain injury; and poorer cognitive performances especially involving the working memory domains were reported (see Review Articles by Dikmen et al., 2009; Dunning et al., 2016; Phillips et al., 2017). Research on cognitive deficits in traumatic brain injury has been extensive due to the debilitating conditions brought upon an individual daily life after the injury. Traumatic brain injuries (TBI) refer to accidental damage to the brain after being hit by an object or following rapid acceleration or deceleration (Farrer, 2017). These accidents include falls, assaults, or automobile accidents and patients with TBI can be then categorized into three groups; (1) mild TBI with GCS – Glasgow Coma Scale – score of 13–15; (2) moderate TBI with GCS score of 9–12; and (3) severe TBI with GCS score of 3–8 (Farrer, 2017). In a recently published meta-analysis that specifically looked at working memory impairments in patients with moderate to severe TBI, patients displayed reduced cognitive functions in verbal short-term memory in addition to verbal and visuospatial working memory in comparison to control groups (Dunning et al., 2016). It was also understood from the analysis that the time lapse since injury and age of injury were deciding factors that influenced these cognitive deficits in which longer time post-injury or older age during injury were associated with greater cognitive decline (Dunning et al., 2016).

Nonetheless, it is to be noted that such findings relating to age of injury could not be generalized to the child population since results from the pediatric TBI cases showed that damage could negatively impact developmental skills that could indicate a greater lag in cognitive competency as the child’s frontal lobe had yet to mature (Anderson and Catroppa, 2007; Mandalis et al., 2007; Nadebaum et al., 2007; Gorman et al., 2012). These studies all reported working memory impairment of different domains such as attentional control, executive functions, or verbal and visuospatial working memory in the TBI group, especially for children with severe TBI (Mandalis et al., 2007; Nadebaum et al., 2007; Gorman et al., 2012). Investigation of whether working memory deficits are domain-specific or -general or involve one or more mechanisms, has yielded inconsistent results. For example, Perlstein et al. (2004) found that working memory was impaired in the TBI group only when complex manipulation such as sequential coding of information is required and not accounted for by processing speed or maintenance of information, but two teams of researchers (Perbal et al., 2003; Gorman et al., 2012) suggested otherwise. From their study on timing judgments, Perbal et al. (2003) concluded that deficits were not related to time estimation but more on generalized attentional control, working memory and processing speed problems; while Gorman et al. (2012) also attributed the lack of attentional focus to impairments observed during the working memory task. In fact, in a later study by Gorman et al. (2016), it was shown that processing speed mediated TBI effects on working memory even though the mediation was partial. On the other hand, Vallat-Azouvi et al. (2007) reported impairments in the working memory updating domain that came with high executive demands for TBI patients. Also, Mandalis et al. (2007) similarly highlighted potential problems with attention and taxing cognitive demands in the TBI group.

From the neuroscientific perspective, hyper-activation or -connectivity in the working memory circuitry was reported in TBI patients in comparison with healthy controls when both groups engaged in working memory tasks, suggesting that the brain attempted to compensate for or re-establish lost connections upon the injury (Dobryakova et al., 2015; Hsu et al., 2015; Wylie et al., 2015). For a start, it was observed that participants with mild TBI displayed increased activation in the right prefrontal cortex during a working memory task when comparing to controls (Wylie et al., 2015). Interestingly, this activation pattern only occurred in patients who did not experience a complete recovery 1 week after the injury (Wylie et al., 2015). Besides, low activation in the DMN was observed in mild TBI patients without cognitive recovery, and such results seemed to be useful in predicting recovery in patients in which the patients did not recover when hypoactivation (low activation) was reported, and vice versa (Wylie et al., 2015). This might be suggestive of the potential of cognitive recovery simply by looking at the intensity of brain activation of the DMN, for an increase in activation of the DMN seemed to be superseded before cognitive recovery was present (Wylie et al., 2015).

In fact, several studies lent support to the speculation mentioned above as hyperactivation or hypoactivation in comparison with healthy participants was similarly identified. When sex differences were being examined in working memory functional activity in mild TBI patients, hyperactivation was reported in male patients when comparing to the male control group, suggesting that the hyperactivation pattern might be the brain’s attempt at recovering impaired functions; even though hypoactivation was shown in female patients as compared to the female control group (Hsu et al., 2015). The researchers from the study further explained that such hyperactivation after the trauma acted as a neural compensatory mechanism so that task performance could be maintained while hypoactivation with a poorer performance could have been the result of a more severe injury (Hsu et al., 2015). Therefore, the decrease in activation in female patients, in addition to the observed worse performance, was speculated to be due to a more serious injury sustained by the female patients group (Hsu et al., 2015).

In addition, investigation of the effective connectivity of moderate and severe TBI participants during a working memory task revealed that the VMPFC influenced the ACC in these TBI participants when the opposite was observed in healthy subjects (Dobryakova et al., 2015). Moreover, increased inter-hemispheric transfer due to an increased number of connections between the left and right hemispheres (hyper-connectivity) without clear directionality of information flow (redundant connectivity) was also reported in the TBI participants (Dobryakova et al., 2015). This study was suggestive of location-specific changes in the neural network connectivity following TBI depending on the cognitive functions at work, other than providing another support to the neural compensatory hypothesis due to the observed hyper-connectivity (Dobryakova et al., 2015).

Nevertheless, inconsistent findings should not be neglected. In a study that also focused on brain connectivity analysis among patients with mild TBI by Hillary et al. (2011), elevated task-related connectivity in the right hemisphere, in particular the prefrontal cortex, was consistently demonstrated during a working memory task while the control group showed greater left hemispheric activation. This further supported the right lateralization of the brain to reallocate cognitive resources of TBI patients post-injury. Meanwhile, the study did not manage to obtain the expected outcome in terms of greater clustering of whole-brain connections in TBI participants as hypothesized (Hillary et al., 2011). That said, no significant loss or gain of connections due to the injury could be concluded from the study, as opposed to the hyper- or hypoactivation or hyper-connectivity frequently highlighted in other similar researches (Hillary et al., 2011). Furthermore, a study by Chen et al. (2012) also failed to establish the same results of increased brain activation. Instead, with every increase of the working memory load, increase in brain activation, as expected to occur and as demonstrated in the control group, was unable to be detected in the TBI group (Chen et al., 2012).

Taken all the insightful studies together, another aspect not to be neglected is the neuroimaging techniques employed in contributing to the literature on TBI. Modalities other than fMRI, which focuses on localization of brain activities, show other sides of the story of working memory impairments in TBI to offer a more holistic understanding. Studies adopting electroencephalography (EEG) or diffusor tensor imaging (DTI) reported atypical brainwaves coherence or white matter integrity in patients with TBI (Treble et al., 2013; Ellis et al., 2016; Bailey et al., 2017; Owens et al., 2017). Investigating the supero-lateral medial forebrain bundle (MFB) that innervates and consequently terminates at the prefrontal cortex, microstructural white matter damage at the said area was indicated in participants with moderate to severe TBI by comparing its integrity with the control group (Owens et al., 2017). Such observation was backed up by evidence showing that the patients performed more poorly on attention-loaded cognitive tasks of factors relating to slow processing speed than the healthy participants, although a direct association between MFB and impaired attentional system was not found (Owens et al., 2017).

Correspondingly, DTI study of the corpus callosum (CC), which described to hold a vital role in connecting and coordinating both hemispheres to ensure competent cognitive functions, also found compromised microstructure of the CC with low fractional anisotropy and high mean diffusivity, both of which are indications of reduced white matter integrity (Treble et al., 2013). This reported observation was also found to be predictive of poorer verbal or visuospatial working memory performance in callosal subregions connecting the parietal and temporal cortices (Treble et al., 2013). Adding on to these results, using EEG to examine the functional consequences of CC damage revealed that interhemispheric transfer time (IHTT) of the CC was slower in the TBI group than the control group, suggesting an inefficient communication between the two hemispheres (Ellis et al., 2016). In addition, the TBI group with slow IHTT as well exhibited poorer neurocognitive functioning including working memory than the healthy controls (Ellis et al., 2016).

Furthermore, comparing the working memory between TBI, MDD, TBI-MDD, and healthy participants discovered that groups with MDD and TBI-MDD performed poorer on the Sternberg working memory task but functional connectivity on the other hand, showed that increased inter-hemispheric working memory gamma connectivity was observed in the TBI and TBI-MDD groups (Bailey et al., 2017). Speculation provided for the findings of such neuronal state that was not reflected in the explicit working memory performance was that the deficits might not be detected or tested by the utilized Sternberg task (Bailey et al., 2017). Another explanation attempting to answer the increase in gamma connectivity in these groups was the involvement of the neural compensatory mechanism after TBI to improve performance (Bailey et al., 2017). Nevertheless, such outcome implies that behavioral performances or neuropsychological outcomes might not always be reflective of the functional changes happening in the brain.

Yet, bearing in mind that TBI consequences can be vast and crippling, cognitive improvement or recovery, though complicated due to the injury severity-dependent nature, is not impossible (see Review Article by Anderson and Catroppa, 2007; Nadebaum et al., 2007; Dikmen et al., 2009; Chen et al., 2012). As reported by Wylie et al. (2015), cognitive improvement together with functional changes in the brain could be detected in individuals with mild TBI. Increased activation in the brain during 6-week follow-up was also observed in the mild TBI participants, implicating the regaining of connections in the brain (Chen et al., 2012). Administration of certain cognitively enhancing drugs such as methylphenidate was reported to be helpful in improving working memory performance too (Manktelow et al., 2017). Methylphenidate as a dopamine reuptake inhibitor was found to have modulated the neural activity in the left cerebellum which subsequently correlated with improved working memory performance (Manktelow et al., 2017). A simplified summary of recent studies on working memory and TBI is tabulated in **Table 4**.

**Table 4:** Working memory (WM) studies in the TBI group.

Authors	Target groups	WM task	Neuroimaging modality	Outcome variables
Bailey et al., 2017	MDD; MDD-TBI; TBI	Sternberg task	EEG	Functional gamma connectivity
Chen et al., 2012	Mild TBI	N-back WM task	fMRI	Brain activations
Dobryakova et al., 2015	Moderate and severe TBI	CapMan task	fMRI	Effective connectivity
Ellis et al., 2016	Pediatric moderate and severe TBI	–	EEG (Visual ERP)	Interhemispheric transfer time
Gorman et al., 2012	Pediatric TBI	CLS; CLS-DT; VSS; VSS-DT	–	Neuropsychological test performances
Hillary et al., 2011	Moderate and severe TBI	N-back WM task	fMRI	Effective connectivity
Hsu et al., 2015	Mild TBI	N-back WM task	fMRI	Brain activations in ROIs
Mandalis et al., 2007	Pediatric moderate and severe TBI	Forward digit span task (WISC-III); TOL; COWAT; The animal fluency test	–	Neuropsychological test performances
Manktelow et al., 2017	Moderate and severe TBI	N-back WM task; Rapid Visual Information Processing Task; Stop Signals Task; TOL	DTI; fMRI	Structural and functional connectivity after methylphenidate administration
Rodriguez Merzagora et al., 2014	Moderate and severe TBI	Verbal n-back WM task	fNIRS	Hemodynamic responses in ROIs
Owens et al., 2017	Moderate and severe TBI	Selective attention tasks; N-back WM task	DTI	Structural connectivity in medial forebrain bundle
Perbal et al., 2003	Severe TBI	Corkin WM Test	–	Neuropsychological test performances
Perlstein et al., 2004	TBI	Visual n-back WM task	fMRI	Brain activations
Treble et al., 2013	Pediatric TBI	CLS-DT; VSS-DT	DTI	Structural connectivity in corpus callosum
Wylie et al., 2015	Mild TBI	N-back WM task	fMRI	Brain activations

*MDD, Major depressive disorder; CLS, Category listening span task; CLS-DT, Category listening span dual-task; VSS, Visuospatial span task; VSS-DT, Visuospatial span dual-task; WISC-III, Wechsler Intelligence Scale for Children-Third Edition-Australian Adaptation; TOL, Tower of London; COWAT, Controlled Oral Word Association Test; EEG, Electroencephalography; fMRI, Functional magnetic resonance imaging; ERP, Event-related potential; DTI, Diffusor tensor imaging; fNIRS, Functional near-infrared spectroscopy; ROIs, Regions of interest.*

## General Discussion and Future Direction

In practice, all of the aforementioned studies contribute to the working memory puzzle by addressing the topic from different perspectives and employing various methodologies to study it. Several theoretical models of working memory that conceptualized different working memory mechanisms or domains (such as focus of attention, inhibitory controls, maintenance and manipulation of information, updating and integration of information, capacity limits, evaluative and executive controls, and episodic buffer) have been proposed. Coupled with the working memory tasks of various means that cover a broad range (such as Sternberg task, n-back task, Corsi block-tapping test, Wechsler’s Memory Scale [WMS], and working memory subtests in the Wechsler Adult Intelligence Scale [WAIS] – Digit Span, Letter Number Sequencing), it has been difficult, if not highly improbable, for working memory studies to reach an agreement upon a consistent study protocol that is acceptable for generalization of results due to the constraints bound by the nature of the study. Various data acquisition and neuroimaging techniques that come with inconsistent validity such as paper-and-pen neuropsychological measures, fMRI, EEG, DTI, and functional near-infrared spectroscopy (fNIRS), or even animal studies can also be added to the list. This poses further challenges to quantitatively measure working memory as only a single entity. For example, when studying the neural patterns of working memory based on Cowan’s processes-embedded model using fMRI, one has to ensure that the working memory task selected is fMRI-compatible, and demands executive control of attention directed at activated long-term memory (domain-specific). That said, on the one hand, there are tasks that rely heavily on the information maintenance such as the Sternberg task; on the other hand, there are also tasks that look into the information manipulation updating such as the n-back or arithmetic task. Meanwhile, the digit span task in WAIS investigates working memory capacity, although it can be argued that it also encompasses the domain on information maintenance and updating-. Another consideration involves the different natures (verbal/phonological and visuospatial) of the working memory tasks as verbal or visuospatial information is believed to engage differing sensory mechanisms that might influence comparison of working memory performance between tasks of different nature (Baddeley and Hitch, 1974; Cowan, 1999). For instance, though both are n-back tasks that includes the same working memory domains, the auditory n-back differs than the visual n-back as the information is presented in different forms. This feature is especially crucial with regards to the study populations as it differentiates between verbal and visuospatial working memory competence within individuals, which are assumed to be domain-specific as demonstrated by vast studies (such as Nadler and Archibald, 2014; Pham and Hasson, 2014; Nakagawa et al., 2016). These test variations undeniably present further difficulties in selecting an appropriate task. Nevertheless, the adoption of different modalities yielded diverging outcomes and knowledge such as behavioral performances, functional segregation and integration in the brain, white matter integrity, brainwave coherence, and oxy- and deoxyhaemoglobin concentrations that are undeniably useful in application to different fields of study.

In theory, the neural efficiency hypothesis explains that increased efficiency of the neural processes recruit fewer cerebral resources in addition to displaying lower activation in the involved neural network (Vartanian et al., 2013; Rodriguez Merzagora et al., 2014). This is in contrast with the neural compensatory hypothesis in which it attempted to understand diminished activation that is generally reported in participants with TBI (Hillary et al., 2011; Dobryakova et al., 2015; Hsu et al., 2015; Wylie et al., 2015; Bailey et al., 2017). In the diseased brain, low activation has often been associated with impaired cognitive function (Chen et al., 2012; Dobryakova et al., 2015; Wylie et al., 2015). Opportunely, the CRUNCH model proposed within the field of aging might be translated and integrated the two hypotheses here as it suitably resolved the disparity of cerebral hypo- and hyper-activation observed in weaker, less efficient brains as compared to healthy, adept brains (Reuter-Lorenz and Park, 2010; Schneider-Garces et al., 2010). Moreover, other factors such as the relationship between fluid intelligence and working memory might complicate the current understanding of working memory as a single, isolated construct since working memory is often implied in measurements of the intelligence quotient (Cowan, 2008; Vartanian et al., 2013). Indeed, the process overlap theory of intelligence proposed by Kovacs and Conway (2016) in which the constructs of intelligence were heavily scrutinized (such as general intelligence factors, *g* and its smaller counterparts, fluid intelligence or reasoning, crystallized intelligence, perceptual speed, and visual-spatial ability), and fittingly connected working memory capacity with fluid reasoning. Cognitive tests such as Raven’s Progressive Matrices or other similar intelligence tests that demand complex cognition and were reported in the paper had been found to correlate strongly with tests of working memory (Kovacs and Conway, 2016). Furthermore, in accordance with such views, in the same paper, neuroimaging studies found intelligence tests also activated the same fronto-parietal network observed in working memory (Kovacs and Conway, 2016).

On the other hand, even though the roles of the prefrontal cortex in working memory have been widely established, region specificity and localization in the prefrontal cortex in relation to the different working memory domains such as manipulation or delayed retention of information remain at the premature stage (see Review Article by D’Esposito and Postle, 2015). It has been postulated that the neural mechanisms involved in working memory are of high-dimensionality and could not always be directly captured and investigated using neurophysiological techniques such as fMRI, EEG, or patch clamp recordings even when comparing with lesion data (D’Esposito and Postle, 2015). According to D’Esposito and Postle (2015), human fMRI studies have demonstrated that a rostral-caudal functional gradient related to level of abstraction required of working memory along the frontal cortex (in which different regions in the prefrontal cortex [from rostral to caudal] might be associated with different abstraction levels) might exist. Other functional gradients relating to different aspects of working memory were similarly unraveled (D’Esposito and Postle, 2015). These proposed mechanisms with different empirical evidence point to the fact that conclusive understanding regarding working memory could not yet be achieved before the inconsistent views are reconciled.

Not surprisingly, with so many aspects of working memory yet to be understood and its growing complexity, the cognitive neuroscience basis of working memory requires constant research before an exhaustive account can be gathered. From the psychological conceptualization of working memory as attempted in the multicomponent working memory model (Baddeley and Hitch, 1974), to the neural representations of working memory in the brain, especially in the frontal regions (D’Esposito and Postle, 2015), one important implication derives from the present review of the literatures is that working memory as a psychological construct or a neuroscientific mechanism cannot be investigated as an isolated event. The need for psychology and neuroscience to interact with each other in an active feedback cycle exists in which this cognitive system called working memory can be dissected at the biological level and refined both empirically, and theoretically.

## Conclusion

In summary, the present article offers an account of working memory from the psychological and neuroscientific perspectives, in which theoretical models of working memory are presented, and neural patterns and brain regions engaging in working memory are discussed among healthy and diseased brains. It is believed that working memory lays the foundation for many other cognitive controls in humans, and decoding the working memory mechanisms would be the first step in facilitating understanding toward other aspects of human cognition such as perceptual or emotional processing. Subsequently, the interactions between working memory and other cognitive systems could reasonably be examined.

## Author Contributions

WC wrote the manuscript with critical feedback and consultation from AAH. WC and AAH contributed to the final version of the manuscript. JA supervised the process and proofread the manuscript.

## Conflict of Interest Statement

The authors declare that the research was conducted in the absence of any commercial or financial relationships that could be construed as a potential conflict of interest. The reviewer EB and handling Editor declared their shared affiliation.
